# A Qualitative Exploration of PrEP Interests, Barriers, and Interventions Among Black and Latina Cisgender Women in the U.S.

**DOI:** 10.1007/s10508-023-02712-5

**Published:** 2023-10-05

**Authors:** Liesl A. Nydegger, Heran Kidane, Sabrina Benitez, Mandy Yuan, Kasey R. Claborn

**Affiliations:** 1 Department of Health, Behavior and Society, Bloomberg School of Public Health, Johns Hopkins University, Hampton House, 624 N. Broadway Street, Baltimore, MD 21205 USA; 2 Department of Kinesiology and Health Education, University of Texas at Austin, Austin, TX USA; 3 Department of Molecular Biosciences, University of Texas at Austin, Austin, TX USA; 4 School of Human Ecology, University of Texas at Austin, Austin, TX USA; 5 School of Social Work, University of Texas at Austin, Austin, USA

**Keywords:** HIV/AIDS, Pre-exposure prophylaxis (PrEP), Black, African American women, Latina, Hispanic women

## Abstract

Black and Latina cisgender women (BLCW) are disproportionally affected by HIV, particularly in the southern U.S. In Austin, Texas, Black women contract HIV 18.4 times more and Latinas 2.6 times more compared to White women. Pre-exposure prophylaxis (PrEP) is a medication that prevents contracting HIV; however, PrEP adoption among women is low. The current qualitative study aimed to explore PrEP awareness, interest, preferred PrEP administration methods, barriers to PrEP adoption, and future programs to increase PrEP adoption and adherence among BLCW. A total of 18 BLCW at high risk for HIV were enrolled. Participants completed 3 semi-structured interviews across 3 months. Interviews were transcribed verbatim, coded, and analyzed using thematic content analysis. Results demonstrated that BLCW had low PrEP awareness, high initial PrEP interest, and were interested in a long-acting injectable form of PrEP. Barriers to PrEP adoption included concerns regarding side effects, concerns about adherence to the currently available daily pill, and difficulty with insurance. Participants proposed different ideas for interventions, including support groups, education, community-level programs, and structural interventions. Future studies should focus on increasing PrEP awareness and HIV risk, consider alternative forms of PrEP, educate providers and medical staff on PrEP, and consider tailored interventions to reduce HIV risk among BLCW.

**Supplementary Information:**

The online version contains supplementary material available at 10.1007/s10508-023-02712-5.

## Introduction

HIV is a public health crisis among Black and Latina cisgender women (BLCW) who live in the South. In Austin, Texas, Black women contract HIV 18.4 times and Latinas contract HIV 2.6 times that of White women (AIDSVu, 2021). Texas is 1 of 8 states with the highest rate of new diagnoses and Travis County, Texas, where Austin is located, is 1 of 48 US counties that is a geographic hotspot with extremely high HIV diagnosis rates (Centers for Disease Control and Prevention [CDC], 2020; U.S. Department of Health & Human Services, 2019).

### HIV Risk

The disparities in HIV risks and outcomes impacting BLCW are not mutually exclusive from the numerous, multidimensional disparities experienced by BLCW. Disparities negatively harming BLCW, such as relationship dynamics, limited Black men available to date, and intimate partner violence (IPV), pose their own public health crisis as well as exacerbate the risk of HIV among BLCW (Bowleg et al., 2015, 2021; Gonzalez-Guarda et al., 2017; Ibañez et al., 2017; Joe et al., 2020; Leemis et al., 2022; Oser et al., 2017; Stockman et al., 2015). Previous research identified that BLCW’s relationships with men increase women’s risk and perceive risk for HIV due to actual and perceived infidelity of male partners (Gonzalez-Guarda et al., 2017; Ibañez et al., 2017; Oser et al., 2017) and double standards, such as believing Black women transmit HIV/STIs without considering their own ability to transmit HIV/STIs to their female sexual partner (Bowleg et al., 2015, 2021) or that Black women must remain sexually exclusive to their male partner but males do not need to remain sexually exclusive (Bowleg et al., 2021). There is also a structural mismatch for BLCW to find male partners due to concerns regarding infidelity (Ibañez et al., 2017; Oser et al., 2017) and the overincarceration of Black men (Oser et al., 2017). These considerations emphasize BLCW’s risk for HIV and shapes the need to link BLCW to PrEP.

IPV is a significant public health issue and is linked to various negative health outcomes, such as HIV, sexually transmitted infections (STIs), mental health symptoms, and substance use disorders (Campbell, 2002; Gilbert et al., 2023; Leemis et al., 2022; Phillips et al., 2014). Women experiencing IPV have been found to have higher rates of HIV and engage in behaviors that put them at higher risk for HIV compared to women who do not experience IPV (Gilbert et al., 2000; Seth et al., 2010, 2015). Previous literature identified indirect and direct mechanisms for increased HIV due to IPV (Jeffers et al., 2022). Trauma and abrasion due to sexual assault injuries are associated with increased HIV risk (Draughon, 2012), and IPV by an abusive partner such forced sex, birth control sabotage, condomless nonconsensual sex, and controlling behaviors around sexual activity and contraception increases risk for HIV (Bergmann & Stockman, 2015). IPV is associated with decreased condom use and challenges with safe sex conversations due to fear of violence and power imbalances (El-Bassel et al., 2011; Rountree et al., 2016).

Substance use is also a risk factor for contracting HIV. Previous studies found a link between alcohol and substance use and risky sexual behaviors, such as condomless sex, multiple partners, and partners who engage in behaviors that put them at higher risk for HIV (Epperson et al., 2009; Metrik et al., 2016; Seth et al., 2011). In addition, as the reported frequency of substance use increases, HIV risk has been found to increase among young adults (Patrick et al., 2012). IPV and substance use both independently pose a risk for HIV as well as a syndemic public health issue as together they exacerbate the risk of HIV among BLCW (Batchelder et al., 2016; Gilbert et al., 2000; Nydegger et al., 2021b; Willie et al., 2021).

### PrEP

Pre-exposure prophylaxis (PrEP) is a medication that prevents one from contracting HIV (CDC, 2021b). PrEP is available as a once daily pill and is effective among individuals who use it daily (CDC, 2021a; Collier et al., 2018; Sheth et al., 2016). In 2018, only 7% of women eligible and who could benefit from were prescribed PrEP (CDC, 2021c). PrEP uptake among women is extremely low, particularly in the South (Siegler et al., 2018), which is concerning given BLCW’s risk for HIV.

### Lack of PrEP Awareness Among Black and Latina Cisgender Women

Several studies found that there was low PrEP awareness among BLCW (Auerbach et al., 2015; Bradley et al., 2019; Chapman Lambert et al., 2018; Collier et al., 2017; Hill et al., 2021; Hirschhorn et al., 2020; Patel et al., 2019), especially when compared White women (Raifman et al., 2019), even though Truvada for PrEP was approved by the U.S. Food and Drug Administration (FDA) in 2012 (HIV.gov, 2012). Participants who were aware of PrEP only had name recognition or incorrect/incomplete knowledge (Hill et al., 2021). In another study, after participants disclosed that they had not heard of PrEP, they were enthusiastic about learning more about PrEP (Collier et al., 2017).

### PrEP Interest Among Black and Latina Cisgender Women

A number of studies among BLCW reported that a majority of participants were interested in PrEP (Bond & Gunn, 2016; Bradley et al., 2019; Hill et al., 2021; Patel et al., 2019; Sales et al., 2018). Participants interested in PrEP identified themselves as both high and low risk (Hill et al., 2021). A study among young Black women found that perceived HIV risk was only marginally associated with PrEP interest (Mangum et al., 2022). Another study among Black women reported that participants were interested in PrEP because it is female-controlled, provides protection for women with risky sexual partners, provides protection for serodiscordant couples, and is empowering (Bond & Gunn, 2016).

### PrEP Administration Method Interests

The two available daily medications for PrEP are Truvada and Descovy (CDC, 2021a). Although the FDA approved both daily medications, Truvada is the only FDA-approved daily PrEP medication specifically for cisgender women, categorized as those who participate in vaginal sex (CDC, 2022). Descovy’s effectiveness has not been evaluated in populations who have receptive vaginal sex (US FDA, 2019). While Apretude is now FDA approved as an injection form of PrEP (US FDA, 2021), this was not available during data collection; only daily pills were FDA approved at the time. One study among Black women found that taking a daily pill would be cumbersome (Chandler et al., 2020). Several studies reported injection preference over daily pills (Bekker et al., 2020; Philbin et al., 2020, 2021; Tolley et al., 2019). One study conducted a non-experimental study among 563 women who were predominantly Black (35.7%) or White (33.7%). Preferred PrEP administration method tended to coincide with familiarity and successful birth control methods (Calabrese et al., 2020).

### Barriers to PrEP

Barriers to PrEP adoption include low PrEP awareness, access, cost, stigma, medical mistrust, insurance, transportation, childcare, a daily pill form, and low perceived risk (Auerbach et al., 2015; Bond & Gunn, 2016; Bradley et al., 2019; Chapman Lambert et al., 2018; Collier et al., 2017; Flash et al., 2017; Hill et al., 2021; Nydegger et al., 2021a, 2021b; Ojikutu et al., 2018). PrEP-related stigma was associated with decreased PrEP interest; PrEP intentions were only high when participants reported both low PrEP-related stigma and low anticipated PrEP disapproval by others (Calabrese et al., 2018). Stigma also includes healthcare provider stigma and reported judgement when showing interest in using PrEP (Mayer et al., 2020). A review found that there was low perceived HIV risk among women, serving as a barrier to PrEP adoption, yet one study reported that over half of women who seroconverted during a PrEP trial reported low perceived HIV risk (Chapman Lambert et al., 2018). Low perceived risk among Black individuals was the predominate reason for participants’ lack of interest in PrEP (Ojikutu et al., 2018). Furthermore, studies have shown that many providers lack the knowledge to discuss PrEP with patients and training for PrEP implementation (Jackson et al., 2022; Seidman et al., 2016; Wilson et al., 2021). With this knowledge gap, providers feel discomfort discussing and prescribing PrEP, which serves as an obstacle for patients to receive information about PrEP (Wilson et al., 2021).

### PrEP Implementation Strategies

Studies have addressed PrEP implementation strategies among BLCW. One study assessing Black women’s attitudes toward potential PrEP recruitment strategies suggest distributing PrEP information in spaces BLCW congregate, using BLCW on PrEP advertisements, and advertising PrEP via social media and text messages. The same study suggests expanding the number of providers offering PrEP by facilitating provider training, offering and delivering PrEP during established provider visits to strengthen the clinician-patient relationship, and offering various delivery methods of PrEP (Arnold et al., 2022). Among South African women that were offered on-site PrEP access, HIV seroconversion decreased by half, supporting the success of PrEP implementation efforts (Donnell et al., 2021).

### Study Purpose

There is an urgent need to better engage BLCW with PrEP, which includes programs to educate BLCW about PrEP, increase risk awareness, and improve knowledge and training of providers who work with BLCW (Kwakwa et al., 2018; Ojikutu et al., 2018). Few qualitative studies have linked participants with PrEP services and then followed them over time to explore facilitators and barriers to PrEP adoption (Nydegger et al., 2021a, 2021b), particularly among BLCW. Additionally, few PrEP-related studies explore potential programs to increase PrEP adoption among BLCW. This study sought to elicit PrEP awareness and interest, PrEP administration method preferences, barriers to PrEP adoption, and potential programs to increase PrEP adoption and adherence among BLCW over time. Participants were interviewed three times across three months to allow time for PrEP adoption, changes in PrEP interest, and determine real-world barriers to PrEP adoption.

## Method

### Participants

We conducted semi-structured, in-depth interviews with 18 BLCW three times over three months from May 2018 to November 2019. We planned on recruiting 20 participants but reached data saturation at 18 participants and thus stopped recruiting. Initial eligibility criteria were determined over the phone prior, which included being at least 18 years old, BLCW, at least one child under 18 years old, unprotected vaginal or anal sex with a cisgender man in the past 30 days, unknown or HIV-negative status, and spoke fluent English or Spanish. If participants were initially eligible, we scheduled an in-person interview. To ensure participants were at risk for HIV, additional eligibility criteria included at least two of the following risk factors: IPV in the past three months; substance misuse in the past 30 days; transactional sex in the past three months; multiple sex partners in the past three months; or their partner(s) had multiple sex partners in the past three months.

### Procedure

Purposive and incentivized snowball sampling occurred in Travis County, TX at community organizations, community clinics, and by participant referral. Participants were given the option to refer up to three eligible participants, receiving $10 for each referral. Interviews occurred in a private place in which the participant preferred which included their homes, parks, or a private interview room at LAN’s office.

Participants were consented and additional eligibility criteria was determined via an audio-recorded structured interview (i.e., at least two of the following: IPV, substance misuse, transactional sex, multiple sex partners, and/or a partner with multiple sex partners). Eligible participants continued with the in-depth, semi-structured interview and were interviewed again at one and three months. We partnered with local community healthcare clinics to refer participants; after completing the baseline interview, participants were asked if they were interested in being screened for PrEP by a medical provider. Participants who expressed interest signed a Health Insurance Portability and Accountability Act (HIPAA) waiver for the clinic to share their medical records with the research team during the participants’ engagement with the study. Participants who chose not to sign the HIPAA waiver remained in the study.

Participants were contacted every 2 weeks in between interviews to increase study retention (see Supplement A for scripts). Participants were compensated increasing amounts of $25, $30, and $40 for interviews; those who travelled to LAN’s office were reimbursed $5 and those who needed childcare during interviews were reimbursed $15. All interviews were conducted in English (*n* = 17) or Spanish (*n* = 1) by female interviewers. LAN who was trained in qualitative methods and conducted previous studies using these methods (McDaniel et al., 2023; Nydegger & Claborn, 2020; Nydegger et al., 2021a, 2021b) conducted a majority of interviews; the remaining interviews were conducted by research assistants trained by LAN. Interviews were between 30 min and 3.3 h *(M* = 1.4 h), with interview 1 averaging 1.88 h (range 1.1–3.3 h), interview 2 averaging 0.97 h (range 0.53–1.47 h), and interview 3 averaging 1.35 h (range 0.5–2.45 h)*.* Interviews 1 and 3 included the most questions, hence they tended to be longer. Interview lengths also differed based on participants’ disclosure of different behaviors; for example, if a participant did not engage in any substance use prior to the relevant interview, all substance use-related questions were not asked. Also, some participants reported situations they experienced without prompting; statements that were relevant to the study (e.g., long story about a partner who cheated) were not interrupted. Participants were informed about the sensitive nature of many of the questions so that they could choose the most appropriate interview location.

### Measures

Interview guides explored daily life experiences, relationship and romantic experiences, IPV, substance misuse, HIV knowledge, PrEP knowledge, PrEP attitudes, and PrEP interest (see Supplement A). The present manuscript focused on discussions related to HIV and PrEP including PrEP interest, attitudes, and future programs to increase PrEP uptake and adherence. These factors were explored across three interviews to ascertain any changes and to provide participants time to adopt PrEP if they were interested.

### Data Analysis

Interviews were transcribed verbatim. First, we developed a preliminary codebook based on the interview guides and review of the literature. The codebook was refined across multiple iterations (7 versions total) via team coding (two coders) of baseline and follow-up interviews (see Supplement B), which were coded in NVivo. After finalizing the codebook, two coders double coded three transcripts (one from each interview) with 96% reliability. Discrepancies were discussed and ultimately agreed upon. With the present study focused on PrEP, three coders identified codes that could be collated into themes, determined themes that arose from reading transcripts line-by-line, and further refined themes until consensus was reached (Braun & Clarke, 2006). Participants’ interviews (total *n* = 47) were analyzed sequentially to explore and identify changes in PrEP interest, attitudes, uptake, and adherence. Participant ID numbers are used to protect participants’ privacy.

## Results

See Table 1 for demographic and variables of interest.

**Table 1:** Demographics and variable of interest by PrEP interest

	Interested *n* = 11 *n* (%)	Decreased Interest *n* = 5 *n* (%)	Uninterested *n* = 2 *n* (%)	Total *n* = 18 *n* (%)
*PrEP awareness*				
Unaware	8 (72.7)	3 (60.0)	1 (50.0)	12 (66.7)
Limited/incorrect	1 (9.1)	1 (20.0)	1 (50.0)	3 (16.7)
Aware	2 (18.2)	1 (20.0)	0 (0.0)	3 (16.7)
*PrEP administration*^a^				
Pill	4 (36.4)	3 (60.0)	1 (50.0)	8 (44.4)
Vaginal ring	2 (18.2)	1 (20.0)	0 (0.0)	3 (16.7)
Injection	5 (45.5)	2 (40.0)	1 (50.0)	8 (44.4)
Implant	3 (27.3)	1 (20.0)	0 (0.0)	4 (22.2)
Other	0 (0.0)	1 (20.0)	0 (0.0)	1 (5.6)
*PrEP barriers*^a^				
Insurance	5 (45.5)	1 (20.0)	0 (0.0)	5 (27.8)
Side effects	8 (72.7)	2 (40.0)	2 (100.0)	12 (66.7)
Pill adherence	5 (45.5)	2 (40.0)	2 (100.0)	9 (50.0)
Medical mistrust	2 (18.2)	1 (20.0)	1 (50.0)	4 (22.2)
*Perceived risk*				
None	4 (36.4)	0 (0.0)	0 (0.0)	4 (22.2)
Low	0 (0.0)	5 (100.0)	1 (50.0)	6 (33.3)
Moderate	5 (45.5)	0 (0.0)	0 (0.0)	5 (27.8)
High	2 (18.2)	0 (0.0)	1 (50.0)	3 (16.7)
*HIV/PrEP stigma*				
Concerned	2 (18.2)	1 (20.0)	2 (100.0)	5 (27.8)
Unconcerned	9 (81.9)	4 (80.0)	0 (0.0)	13 (72.2)

^a^Not mutually exclusive

### PrEP Awareness

Two–thirds of the participants were unaware of PrEP; of those who were aware of PrEP, half had limited or incorrect knowledge of PrEP (i.e., one participant had only heard of PrEP from the recruitment flyer, one thought it was an injection (which was unavailable at the time), several only saw advertisements that they did not relate to and one thought it was only for men who have sex with men (MSM), and one thought it was an immunization, which she was against all immunizations for both her and her children). Participants who expressed interest in PrEP reported being more unaware of PrEP prior to the study than participants who were uninterested in PrEP throughout the study.

### PrEP Interest

Three differing interest groups emerged from the data. A majority of participants were interested in PrEP throughout the study, followed by some participants who expressed initial interest in PrEP and then reported decreased interest throughout the study, and a couple participants who were not interested in PrEP. While this was an unexpected finding, particularly participants with changing interests in PrEP (i.e., decreased PrEP interest group), this emerged from the data during coding. Most of the participants who recognized their risk of HIV infection were more interested in PrEP. When asked if she would consider taking PrEP, participant 204 enthusiastically said yes and recognized her risk for HIV (see Table 2, 1.a). Participant 244 shared her thoughts on PrEP as a method of HIV prevention (see Table 2, 1.b).

**Table 2:** Participant quotes by theme

Themes	Quotes
1. PrEP interest	a. Yeah because I’m sexually active and I mess with married men. I’m not in a steady relationship. (204, Interview 1, Interested in PrEP, 44, Black)
	b. It’s a good thing, yeah. Especially with preventing so much HIV, with a once a day pill, something like that, I’m saying it should—that’s something easy to already been out there, you know? (244, Interview 1, Interested in PrEP, 30, Black)
	c. No one wants to put something in their body that could potentially become HIV because in some weird way it’s how it kind of feels…I’m scared to take that pill…Long story short, that big boy [oldest son] in there, he messed it up for the whole world with me and [inaudible], I forgot the name of it right now. But he got a shot that did things to him and caused problems. I don’t let my kids get shots. I know people don’t agree with that, people don’t maybe understand. But there’s a lot of studies of kids with and without and the kids without seem to surpass every type of thing, whether it’s academic, physical, out-live, without getting shots. (240, Interview 1, Uninterested in PrEP, 32, Black)
	d. Probably so, and not saying that I’m at risk or anything because I don’t feel like I’m going to be at risk. (210, Interview 1, Decreased PrEP Interest, 31, Black)
	e. Why should I take a preventative if number one, I don’t think that that affects me and my life? Number two, even if I was changing partners, I wouldn’t be thinking I’m going to come in contact with somebody that has HIV. The cost, probably number one, it would be the cost. Then number two, just seems like a way for pharmaceutical company to make money. (236, Interview 1, Uninterested in PrEP, 47, Latina)
2. Perceived HIV Risk	a. He was really free balling. Really just out there free with his dick. Like he did not care who he slept with. If you thought he was handsome or he thought you looked good and you was willing to have sex right then and there he was with it. (228, Interview 1, Interested in PrEP, 32, Black)
3. Stigma	a. I think a lot of people nowadays think that you can only get it if you’re gay. (224, Interview 1, Interested in PrEP, 34, Black) b. I could see the gay community [being interested in PrEP] because they are very promiscuous and that is their lifestyle. (236, Interview 3, Uninterested in PrEP, 47, Latina)
4. PrEP Administration Preferences	a. I would rather have an injection because I don’t have to worry about taking that pill. (204, Interview 1, Interested in PrEP, 44, Black)
	b. Taking a pill a day, it just like reminds you, get yourself up, get your pill. It’s like you’re getting up, you go to put your clothes, we’ve got to change. I think it would be something that’s easy to take. (244, Interview 1, Interested in PrEP, 30, Black)
	c. I mean you don’t have to keep on going back and forth. You can save transportation, trips, whatever or gas. (206, Interview 1, Interested in PrEP, 24, Latina)
	d. That’s why I never did any kind of implant birth control either because of people that I’ve seen with the funky scars on their arm, I’m like, “No, okay, no I don’t want to do that.” (241, Interview 1, Interested in PrEP, 39, Latina)
	e. Because what if you’d be on your period? I mean like, because couldn’t you get bacteria or something like, I don’t know. Those ring, I mean I’m just not with the leaving stuff in the vag. Or what if break? What if it could puncture you or something? (204, Interview 1, Interested in PrEP, 44, Black)
5. Barriers to PrEP	a. Well, it depends on what the side effects are. That’s all I’m worried about. (230, Interview 1, Interested in PrEP, 30, Latina)
	b. Well it’s not–so it’s a priority in my life, I know I’m not going to sleep with people that have HIV, I just, I know it–or be protected and I used condoms. I mean now I know, I’m more–you know more sexually like stable when it comes to that, those type of things. (242, Interview 1, Decreased PrEP Interest, 21, Black)
	c. ‘Cause I didn’t have insurance and then I didn’t have cash at that time to actually just, but um, with the MAC card, it’ll cover it, so I don’t have to, I’m like, “yeah, I’ll just do it that way” and I need insurance anyway. (228, Interview 2, Interested in PrEP, 32, Black)
6. Interventions	a. Chatting with you, I think that, what you all are doing is, is really helpful and beneficial to people. Like I’m not going to say like, well I feel like if, if there was like a, seminar or not like a seminar but you know kind of like a group study…Like where you can introduce it [PrEP] to people—or at least talk to them about [it] so that they can get educated about it. You know, just to spread the word (210, Interview 2, Decreased PrEP Interest, 31, Black)
	b. When asked what made it difficult to get on PrEP, the participant responded “Not being white. Back in those days they had segregation, and they still have segregation, which I think is ridiculous. By not being white, you can’t get on the PrEP. You’ll have to suffer, basically… I’m not working… Help us get jobs. (216, Interview 3, Interested in PrEP, 24, Black)

Among participants uninterested or with decreasing interest in PrEP, most participants reported low perceived HIV risk, a few were concerned about HIV/PrEP stigma, some mentioned a fear of potential PrEP side effects, and most mentioned the inability to adhere to the PrEP medication schedule. Participant 240 shared her concerns regarding PrEP (see Table 2, 1.c). Some of these participants had incorrect knowledge about PrEP. One participant reported that she did not feel at risk after she was asked if she would take PrEP (see Table 2, 1.d). Participant 236 shared her reasons for not believing she needed PrEP (see Table 2, 1.e). After asking participants if anyone they know may be interested in PrEP, several participants mentioned that people they know would be interested in PrEP.

### Perceived HIV Risk

Over half the participants viewed their risk for HIV as none-to-low (see Table 1). Although, a majority of participants interested in PrEP throughout the study viewed their risk for HIV as moderate-to-high. Participants who viewed themselves as moderate-to-high risk for HIV reported their risk due to having multiple sexual partners or partners who could potentially be cheating. Participant 228 felt at risk for HIV with her husband who she knew was cheating (see Table 2, 2.a). All participants in the decreased PrEP interest group viewed themselves as low risk even though throughout the study most engaged in at-risk behaviors such as having multiple sex partners and engaging in condomless sex.

Interestingly, in the uninterested group, one participant viewed herself at high-risk for HIV because of having condomless sex while the other participant perceived that she was at low risk.

### Perceived HIV- and PrEP-Related Stigma

Most participants from both the interested and decreased PrEP interest groups were not concerned about HIV- or PrEP-related stigma, but many discussed how society in general tends to equate HIV only to MSM (see Table 2, 3.a). Both uninterested participants reported concern regarding HIV- and PrEP-related stigma. For instance, one participant mentioned several times that she only views PrEP to be of interest to MSM (see Table 2, 3.b). This was surprising given that she found that her fiancé was using a popular hook-up app used among MSM and he was having sex with her male neighbor. While she did get tested for HIV and other STIs, she did not think she was at risk for HIV or needed to consider taking PrEP.

### PrEP Administration Preferences

**Fig. 1:**
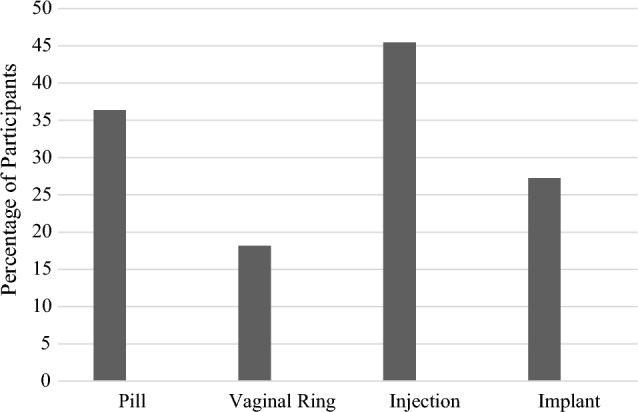
Percentages of PrEP administration preferences among participants interested in PrEP. *Note:* percentages are not mutually exclusive and do not add up to 100%

Overall, most participants reported interest in PrEP as an injection every 2–3 months or a daily pill. Among those interested in PrEP, most participants preferred an injection, followed by several preferring a daily pill, some preferring an annual implant, and a couple preferring a monthly vaginal ring (see Fig. 1). Most participants in the decreased interest group tended to prefer the daily pill.

Some participants preferred the injection because it is a convenient, easy method to administer PrEP with no concerns about having to keep track of taking PrEP. When asked about her preferred PrEP administration method, participant 204 shared her thoughts on injection versus the pill (see Table 2, 4.a). Several participants were uninterested in the injection because they had a fear of needles or shots. Some participants preferred the pill because of routine and convenience (see Table 2, 4.b). Some participants were uninterested in the pill because of concerns with treatment adherence, such as remembering to take a daily pill and not willing to take pills or medicine. Participant 206 explained her rationale behind her preference for the implant (see Table 2, 4.c). Some participants were uninterested in the implant due to fears of discomfort with insertion of the implant or did not want something implanted. Participant 241 relayed her hesitation for the PrEP implant with her fear of other implants (see Table 2, 4.d). A few participants were interested in PrEP as a vaginal ring because they thought it may be more comfortable. Many participants were uninterested in a vaginal ring due to concerns about the hygiene, comfort, and potential health repercussions of ring insertion. Participant 204 shared her hesitation with the vaginal ring (see Table 2, 4.e). One participant mentioned that she preferred another form of PrEP, such as liquid.

### Barriers to PrEP

Barriers to PrEP interest and adoption included insurance, side effects, concerns regarding pill adherence, and medical mistrust. Most participants interested in PrEP were concerned with side effects, followed by some concerned about treatment adherence and difficulty with insurance. Side effects served as the primary concern for participant 230 (see Table 2, 5.a). Participant 242 perceived low risk for HIV infection that led to concerns with treatment adherence (see Table 2, 5.b). A few participants attempted to attend appointments for PrEP evaluation but were unable to see a provider due to lack of or confusion regarding insurance (see Table 2, 5.c). Ultimately, no participants adopted PrEP during the study.

### Interventions

Participants proposed different ideas for interventions to increase PrEP awareness and use among BLCW at high risk for HIV. Most participants indicated the need for sex education in general among BLCW. While sex education should address HIV and PrEP, several participants also indicated that this education should include topics such as healthy relationship communication and IPV, one indicated including substance use, a couple wanted housing including, one mentioned economic stability, one suggested including transactional sex, including survival sex, a couple of participants indicated including job skills, a couple of participants mentioned stigma related to HIV and PrEP, and several suggested that interventions should also be directed toward adolescents and young adults in high school and college. Several participants preferred one-on-one interventions, many participants preferred group-based or support group-based interventions, and several mentioned that an intervention should include a combination of the two. Two participants suggested using an app to provide information and referrals. A few participants suggested doing community-level interventions such as a health fair for women’s health encouraging HIV testing and partner testing, community BBQ or festival with health-related booths, or a health fair (see Table 2, 6.a). Many participants recommended structural-level interventions such as advertisements, including ones in Spanish for BLCW or have celebrities speak about HIV and PrEP, and interventions that targeted structural factors such as housing, financial instability, and insurance (see Table 2, 6.b). All participants who perceived themselves as moderate- to high-risk for HIV reported a preference for educational programs. Participants suggested locations for a future intervention including apps, community or resource centers, shelters, apartment complexes, clinics, parks, and group homes located in Southeast Austin, East Austin, or Central Austin. Intervention facilitators should include someone living with HIV, counselors, doctors/nurses, obstetrician-gynecologist, social workers, someone taking PrEP, and women who look like BLCW. Incentives were also mentioned to increase participation including bus passes or paid transportation, formula for babies, food, and gift cards. Lastly, when asked about a name for a PrEP program for BLCW, participants suggested using the terms empowerment, PrEP, self-reliance, self-awareness, creating and empowering, prevention for the future, PrEP for life, and something “powerful and warm.”

## Discussion

The present study explored BLCW’s awareness of, interest in, and attitudes toward PrEP. We did this by interviewing BLCW three times across three months to provide time for PrEP adoption after they were linked to PrEP services and explored real-time facilitators and barriers to PrEP adoption. Unfortunately, none of the study participants adopted PrEP so we focused primarily on barriers to PrEP adoption, attitudes toward PrEP, and future interventions. Most participants were unaware of PrEP (Auerbach et al., 2015; Bradley et al., 2019; Chapman Lambert et al., 2018; Collier et al., 2017; Hill et al., 2021; Hirschhorn et al., 2020; Patel et al., 2019). Among participants aware of PrEP, half had limited or incorrect knowledge. As in other studies (Bond & Gunn, 2016; Bradley et al., 2019; Hill et al., 2021; Patel et al., 2019; Sales et al., 2018), a majority of participants were interested in PrEP, mainly because they recognized their risk for contracting HIV (Mangum et al., 2022). Three distinct PrEP interest groups were identified: participants who were interested in PrEP throughout the study, participants with decreasing interest, and participants who were never interested in PrEP. This study identifies the importance of tracking PrEP interest over time as interest may wax and wane. For example, most participants reported initial interest in PrEP. However, as the study continued, some participants (i.e., the decreased PrEP interest group) were not as interested in PrEP as they were initially. Although participants in the decreased PrEP interest group reported engaging in HIV-risk behaviors, all stated that they were at low risk for HIV. This is similar to other studies that found low perceived HIV risk among women who were PrEP-eligible or seroconverted (Chapman Lambert et al., 2018). It is imperative to increase awareness, interest, and perceived HIV risk among BLCW. Future research should consider tracking PrEP interest as it may change over time.

Participants were most interested in an injection, followed by a daily pill, implant, and vaginal ring. The injection was preferred because of concerns with pill adherence. Other studies also found that participants preferred injectable PrEP versus a daily oral pill (Bekker et al., 2020; Philbin et al., 2020, 2021; Tolley et al., 2019). While there are numerous behavioral HIV prevention interventions (e.g., Abrams et al., 2020; Campbell et al., 2011; Chandler et al., 2019; Daniel-Ulloa et al., 2016; Jones et al., 2008; Sapiano et al., 2013), future clinical trials on different PrEP administration methods should include BLCW to advance research, similar to other empirical HIV-related literature focused on BLCW.

Among participants interested in PrEP, the main barriers stated included concerns regarding side effects, concerns about adherence to the currently available daily pill, and difficulty with insurance. These findings echo other studies that also found that barriers to PrEP adoption included PrEP side effects (Hill et al., 2021), taking a daily oral pill (Bond & Gunn, 2016), and insurance (Flash et al., 2017; Hill et al., 2021). Increased PrEP education must be conducted to inform PrEP-eligible individuals of the potential side effects. PrEP administration methods other than a daily oral pill may increase adoption and adherence among BLCW; having a variety of PrEP modalities available for BLCW may further increase adoption and adherence (Arnold et al., 2022). Lastly, providers and clinics need to be educated on the multiple methods of funding PrEP so they are able to provide PrEP to uninsured BLCW.

Future interventions should consider the multidimensional needs and interests of BLCW at risk for HIV. In addition to sexual health education, participants mentioned that PrEP interventions should include topics about factors that could increase HIV risk and access to PrEP, such as intimate partner communication, particularly from a gender- (e.g., Project FIO; Dworkin et al., 2006; Dworking et al., 2007; Ehrhardt et al., 2002; Seal & Ehrhardt, 2004) and race/ethnic-specific perspective (Diallo et al., 2010; Gilbert et al., 2021; Wingood et al., 2011), IPV, substance use, housing, and economic stability. These suggestions align with recommendations (El-Bassel et al., 2011; Jeffers et al., 2022) and interventions (DiClemente et al., 2021; Wingood et al., 2011) in previous studies on multidimensional health promotion and HIV prevention education. Participants in this study, similar to other studies on HIV prevention, indicated an interest in a range of intervention formats (Arnold et al., 2022; Hirschhorn et al., 2020; Pasipanodya et al., 2021) including one-on-one meetings, group sessions, collaborative efforts with organizations and health care providers, and structural changes to improve PrEP availability and access including stable, affordable housing, financial stability, and insurance. While individual-level interventions can benefit BLCW, structural-level interventions that address social determinants of health (Blankenship et al., 2015; Edwards & Collins, 2014) should be incorporated to develop multi-level interventions. Locations of where to host interventions varied between participants, however, an overwhelming majority stated that interventions should take place in community spaces with special considerations for those with children and who experience IPV. Many participants also shared how interventions should be facilitated by those the intervention intends to serve and perhaps an individual who is taking PrEP themselves, highlighting the importance of trust and community regarding PrEP and HIV prevention for BLCW (Pasipanodya et al., 2021; Willie et al., 2022).

### Limitations

Similar to previous research, the current manuscript has its limitations. This qualitative study was exploratory in nature. Consistent with qualitative methods, generalizability is limited as this study was conducted among a small sample of BLCW in Austin, Texas. Future research should incorporate women in other geographic hot spots to explore PrEP-related themes by varying regions. Only one participant completed an interview in Spanish, thus providing a limited perspective from Spanish-speaking BLCW. Participants may have under-reported HIV-risk behaviors due to social desirability effects. However, measures were taken to increase participant comfort with discussing sensitive topics to increase accuracy of self-report. Results are limited to participants willing to take part in a study regarding PrEP. Participants were aware that this study was about PrEP and thus they may have overstated their interest in PrEP. Additionally, participants were asked if they were interested in speaking with a medical professional to learn about PrEP (i.e., dichotomous yes/no question). As such, this question may have led participants to respond “yes,” even if they were not interested in PrEP. This was partially offset by exploring participants’ responses and their actions.

### Conclusions

Future research should include strategies to increase PrEP awareness, increase perceived HIV risk, and explore PrEP administration methods among BLCW who are eligible for PrEP. Multi-level interventions uniquely tailored to BLCW to increase PrEP adoption and adherence should consider utilizing support groups, education, community-level programs (i.e., community event focusing on women’s health), and structural interventions (i.e., housing, income).

### Supplementary Information

Below is the link to the electronic supplementary material.

Supplementary file1 (DOCX 32 KB)

Supplementary file2 (DOCX 26 KB)

## Data Availability

Data are available in the Texas Data Repository: https://dataverse.tdl.org/dataset.xhtml?persistentId=doi:10.18738/T8/X61LJB.

## Code Availability

Available in Supplement B.
