# Supplementary material for: A Qualitative Exploration of PrEP Interests, Barriers, and Interventions Among Black and Latina Cisgender Women in the U.S
Source: Arch Sex Behav. 2023 Oct 5;53(2):771–83. doi: 10.1007/s10508-023-02712-5 (PMC10844362; doi:10.1007/s10508-023-02712-5)
Supplement: Supplementary file 1 — Supplementary file1 (DOCX 32 KB) [file 10508_2023_2712_MOESM1_ESM.docx]

**Supplement A: Interview Guides & Check-In Scripts**

**Interview Guide—Baseline Part 1: Eligibility**

Thank you for agreeing to participate! Some of the first questions we have already discussed briefly but I want to go over everything while we’re recording and ask things in a little more detail. This first part will be to check your eligibility so I will ask you questions about your children, sex partners, condom use, substance use, things like that. It will take about 10 minutes. If you are eligible, we will continue with the rest of the interview. Do you have any questions before we start?

**Sociodemographic**

1. What gender do you identify yourself as?
   1. Cisgender woman 🡪 Next Question
   2. Anything else 🡪 Ineligible
2. How old are you?
   1. 18+ 🡪 Next Question
   2. Less than 18 🡪 Ineligible
3. What race or ethnicity do you most identify with?
   1. Black or Hispanic/Latina 🡪 Next Question
   2. Multiracial (including Black and/or Hispanic/Latina) 🡪 Next Question
   3. Anything else 🡪 Ineligible
4. Do you have any children?
   1. Yes 🡪 Next Question
   2. No 🡪 Ineligible
5. How many do you have?
6. What is/are their ages?
   1. At least 1 under 18 🡪 Next Question
   2. All over 18 🡪 Ineligible
7. Who do your child(ren) live with?
   1. Participant at least 50% of the time 🡪 Next Question
   2. Someone temporarily while they are in shelter but getting custody back soon 🡪 Next Question
   3. Anyone else 🡪 Ineligible
8. Have you had vaginal/anal sex with a man in the past 30 days?
   1. Yes 🡪 Next Question
   2. No 🡪 Ineligible
9. What percentage of the time did you use a condom?
   1. All of the time 🡪 Ineligible
   2. Less than all of the time 🡪 Next Question
10. Have you ever been tested for HIV?
    1. If yes: What were the results of your last test?
       1. Negative 🡪 Next Question
       2. Positive 🡪 Ineligible
       3. Unsure 🡪 Next Question
    2. If no 🡪 Next Question

**Sex & Condom Use**

Next, I want to ask you about your sexual history with a main or steady partner. This could be a husband, boyfriend, lover, or live-in partner.

1. In the past 3 months, has there been one person who you consider to be your main sexual partner?
   1. Yes
   2. No 🡪 Skip to 15
2. In the last 3 months, how many times did you have vaginal or anal sex with your main sexual partner?
3. How many of these times did you use a condom?
4. Do you think your main partner has any other sex partners?
5. Do you know your partner’s HIV status—is he positive, negative, or do you not know?

Now I’m going to ask you about casual sex partners. These are sex partners who are not main, steady partners or sex trading partners, but may be casual friends, or one-night stands.

1. In the past 3 months, has there been anyone who you consider to be a casual sex partner?
2. Yes
3. No 🡪 Skip to 20
4. How many casual sex partners have you had?
5. In the last 3 months, how many times did you have vaginal or anal sex with any casual partner?
6. How many of these times did you use a condom?
7. Do you think your casual sex partner(s) has (have) any other sex partners?
8. What is the HIV status of each of your casual partners? Positive, negative, or do you not know?

**Transactional sex**

Sometimes people have sex with someone because they’re in a rough situation and need money, food, shelter, drugs, or something else.

1. In the past 3 months, have you had sex with someone for money, food, clothing, shelter, drugs, or anything else?

**IPV **Only ask if person has had a main partner in the last 3 months****

Now we’re going to talk about your main partner. No matter how well a couple gets along, there are times when they disagree, get annoyed with the other person, want different things from each other, or just have spats or fights because they are in a bad mood, are tired, or for some other reason. Couples also have many different ways of trying to settle their differences. I’m going to list things that might happen when you have differences. Please let me know if any of these happened to you in the last 3 months with your main partner.

1. You had a sprain, bruise, small cut, or felt pain the next day because of a fight with your partner. (physical)
2. Your partner pushed, shoved, or slapped you. (physical)
3. Your partner punched, kicked, or beat you up. (physical)
4. Your partner destroyed something that belonged to you. (emotional)
5. Your partner threatened to hit you. (emotional/verbal)
6. You went to see a doctor or needed to see a doctor because of a fight with your partner. (physical)
7. Your partner used force (like hitting, holding down, or using a weapon) to make you have sex. (sexual)
8. Your partner insisted on sex when you did not want to. (sexual)
9. Your partner insisted on sex without a condom. (sexual)
10. Your partner forbid you from seeing someone, such as friends or family. (social isolation)
11. Your partner tried to keep you dependent on him for money. (economic)

**[IPV = 1 or more]**

**Substance Use**

Now I’m going to ask you about your alcohol and drug use.

1. How many days in the last 30 days did you drink wine, wine coolers, beer, or liquor? [specify which drink]
2. 1 drink is defined as 1 wine cooler, 5 ounces of wine (about half a cup), 1 shot, or 1 beer. How many drinks [insert glasses of wine, beers, shots depending on their answer] per week do you typically have?
3. When was the last time you had 4 or more drinks on one occasion?
4. Let me know if you used any of the following substances in the past 30 days:
5. Crack. How many times?
6. Cocaine. How many times?
7. Another Stimulant. How many times?
8. Heroin. How many times?
9. Another Opiate. How many times?
10. Marijuana. How many times?
11. Methamphetamine. How many times?

**[problematic substance: alcohol 8+ drinks/week; alcohol 4+ drinks on 1 occasion; 14+ times marijuana; any other substance 1+ times]**

****Only participants who reported two of the following: transactional sex, problematic substance use, IPV, unprotected sex, multiple sex partners, or one of their sex partners has multiple sex partners**

**AND**

**all of the sociodemographic eligibility criteria will be considered eligible****

**Ineligible participants:** Thank you; that is all of the questions I have. Do you have any concerns after answering questions? Unfortunately, you are not eligible to participate in the next portion of the interview. I sincerely appreciate the time you took to answer these questions, and I have $5 for you to thank you for your time and participation. Also, here are some resources for you in case you or someone you know are interested. Thank you!

**Eligible participants:** Thank you, those are all of the initial questions I have for you. Based on your answers, you are eligible to participate in the next part of the interview. Would you like to continue?

**Interview Guide—Baseline Part 2**

Now that we’ve gone through the eligibility portion, we’re going to switch gears a bit. We will actually go back to a lot of those questions we just talked about because I’d like to know more about all of those. Right now I’d like to talk about your day-to-day life.

**Personal Life, Daily Routine, Living Situation, and Economic Situation**

- Describe what your daily life is like.
  - *Where do you usually go?*
  - *What do you usually do?*
  - *Who do you usually see?*
  - *What do you do every day?*
- Describe your current living situation.
  - *Home, apartment, shelter, or moving around or on the street?*
  - *[If housed] Do you live with other people? Describe your relationship with them.*
  - Tell me what you think your living situation will be like in 3 months.
- What are your current sources of income (Family, partner, work, TANF, SNAP, SSI, or other benefits etc.)?
  - Do you ever have difficulty paying bills or making ends meet? Describe what you do in those situations.
- What types of resources and support do you have? This could be relationships with friends, family, or a romantic partner, work friends, groups you attend, or anything else.
- What would you say are your personal strengths?
- What does it mean to you to be a [Black/African American or Hispanic/Latina] woman?
- What are challenges you face as a [Black/African American or Hispanic/Latina] woman?
- What are advantages you have as a [Black/African American or Hispanic/Latina] woman?

**First Sexual Experiences (CSA)**

Next, I’d like to talk about your first sexual experiences.

- Tell me about your first sexual experience, not necessarily intercourse (e.g., touching or playing).
  - *Who initiated this experience?*
  - *How did it happen?*
  - *Did you feel like you had a choice to participate?*
  - *What went into your decision to have this experience with him/her?*
  - *How old were you?*
  - *How old was s/he?*
  - *How did you feel afterward?*
- Tell me about the first time you had vaginal sex with a man.
  - *Who initiated this experience?*
  - *How did it happen?*
  - *Did you feel like you had a choice to participate or not?*
  - *What went into your decision to have sex with him?*
  - *How old were you?*
  - *How old was he?*
  - *Did you use a condom?*
  - *How did you feel afterward?*
- [If previous questions did not divulge molestation or CSA] When you were under 18, did you ever have an unwanted sexual experience?
  - [If yes] Do you mind telling me a bit more about what happened?
  - *Who was the other person involved?*
  - *How old were you at the time?*
  - *How long did this go on for? When did it stop and what was the reason it stopped?*
  - *Did you tell anyone? What was their reaction? How did that make you feel?*

**Relationships**

Now let’s talk about relationships in general.

- What are your thoughts and feelings about relationships and sex?
  - Which partner do you think should initiate sex? Why do you feel that way?
  - Whose responsibility is it to initiate the conversation about safe sex or condom use? Why do you feel that way?
- Tell me about your current (most recent) relationship.
  - *How long have you been (were you) together?*
  - *What are some of the things you find (found) satisfying in the relationship?*
  - *What are some of the problems you’ve had in the relationship?*
- Did/do you use condoms with your partner? Tell me more about that.
  - *Who makes the decision about pregnancy/STD prevention in your relationship?*
- [If they used condoms] Have you ever asked your partner to wear a condom? What was the conversation like?
  - *Do you ever have conflicts over this? What happens if you disagree?*
- Did/do you use birth control with your partner? How did you decide birth control was right for you?
  - *Specify methods of birth control and when, if they stopped*
  - *Who makes the decision about pregnancy prevention in your relationship?*

Now I’m going to ask you about times you and your partner may have not gotten along.

- Tell me about a time when your main partner said something mean to you.
  - *What usually happens (happened) before and after?*
- Does (did) your main partner hurt you physically? Tell me more about that.
  - *What usually happens (happened) before and after?*
- Have you ever had sex with your partner when you told him you really didn’t want to? Tell me more about that.
  - *What usually happens before and after?*
- Tell me about the last time you got in a fight or argument with your main partner.
  - *How did the fight start?*
  - *Where did it happen?*
  - *Was anyone else there?*
  - *What happened during the fight?*
  - *What happened after?*
- Do you have other sex partners in addition to your main partner? [how many]
  - Does your main partner know?
  - Do you use condoms with these other partners? [specify]
  - Who makes the decision about contraception/STD prevention in this sexual partnership?
- Have you ever had sex with anyone else when you didn’t want to? If you are comfortable, please tell me about that situation.
- Have you ever had sex and felt bad about it afterwards? If you are comfortable, please describe that situation.
- How many times have you been pregnant?
  - Was it/were these planned? Tell me about that
  - How many times have you given birth?
    - So you mentioned you had been pregnant X times. Can you share with me what happened during the other Y pregnancies?
  - How many children do you have?
  - What are their ages?
  - How many live with you?
  - How many children do you have custody of?
    - What happened when you lost custody of those children?
  - Describe your relationship with your child(ren)?

**Drug Use**

- Do you drink wine, wine coolers, beer, or liquor? [which of these]
  - 1 drink is defined as 1 wine cooler, 5 ounces of wine (about half a cup), 1 shot, or 1 beer. On days that you drink, how many drinks of [insert what they drink] do you usually have?
  - How many days a week do you usually drink? How many drinks/week do you usually drink?
- Do you smoke weed or use any other drugs?
  - How many days in a month do you usually use [inset drugs]?
- Do you use drugs and alcohol at the same time? Tell me more about that.
- Tell me about when you use alcohol.
  - *What’s your drink of choice?*
  - *What do you feel like before you drink?*
  - *What do you feel like while you’re drinking?*
  - *Who do you use alcohol with? What is your relationship like with them?*
  - *What other things are happening while you’re drinking (e.g., talking, dancing, arguing, sex)?*
- Tell me about when you use [insert drug]. [repeat if they use more than 1 drug]
  - *What do you feel like before you use?*
  - *What do you feel like while you’re high?*
  - *Who do you get high with? What is your relationship like with them?*
  - *What other things are happening while you’re high (e.g., talking, dancing, arguing, sex)?*
- Tell me about a time when you had a drink because you were upset about something that happened with your main partner.
- Tell me about a time you drank because you were upset about something else.
- Tell me about a time you used drugs because you were upset about something that happened with your main partner.
- Tell me about a time used drugs because you were upset about something else.
- How often do you have sex while you are drunk? Tell me about that.
  - *Do you use condoms?*
- How often do you have sex while you are high? Tell me about that.
  - *Do you use condoms?*
- How often do you inject? [If yes] Tell me about that. Where do you get your syringes?
  - Have you ever shared a syringe, or shared or measured drugs using the same syringe? Tell me about when you have done that and with whom.
- A lot of people think about having sex for money, food, or shelter. Have you had similar thoughts?
- Have you ever had sex in exchange for something? Tell me about that.
- Have you ever had sex in order to get alcohol, drugs or money to buy alcohol or drugs? Tell me about that.
- Tell me about a time when you felt like you were using too many drugs or alcohol. What made you feel that way?
  - Did you cut back or reduce your alcohol or drug use? When? What was this like?
- Has anyone close to you ever told you they think you are using too many drugs or alcohol? How do you feel when they say that?

**HIV**

Now I’m going to ask you about HIV.

- What have you heard about how someone gets HIV?
- What have you heard about how someone prevents themselves from getting HIV?
- What have you heard about treatments for HIV?
- What do people in your community think of HIV?
- Is there a difference between what women and men think of HIV in your community?
- Have you ever been in a situation where you felt you were at risk for getting HIV? Tell me about that situation.
  - What prevented you from taking precautions [using a condom/not having sex/not injecting]?

**Health Care**

- Do you currently have health insurance?
  - [If no] What barriers prevent you from getting health insurance?
  - [If yes] What kind of insurance do you have?
    - What is helpful about having insurance?
    - Tell me about any problems you’ve had with your insurance?
- Where do you go if you’re sick or injured?
  - *Urgent care, ER, primary care doctor*
- Do you currently take any medication?
  - When do you take it?
  - What reminds you to take it?
- Other than medication, is there anything that you do for your health that you do every (or almost every) day? Tell me about that.

**PrEP**

- Have you ever heard of PrEP, a medication used to prevent HIV?
  - [If yes] What have you heard about it?
  - *[If yes] Who first told you about PrEP?*
  - *[If yes] What did they tell you about it?*
- Have you ever heard people talking about PrEP, even if you weren’t sure what it was?
  - *Who was it*
  - *What did they say?*
- How much do you think people in your community know about PrEP in general?
  - What do they say?
- [If they’ve heard about PrEP] What do you think about PrEP?

PrEP is a safe medication with very few side effects usually used to treat HIV, however, more recently it’s being taken by people who are HIV-negative to prevent themselves from getting HIV. Right now, the only available medication is a once daily pill. In order to work well, the pill needs to be taken every day.

- After hearing that, what are your thoughts about PrEP?
  - Is this a pill you would consider taking? Tell me more about that.
- What concerns do you think you’d have about taking PrEP?
- If you chose to take it, would you want to tell your main partner you’re taking PrEP? Do you think you would end up telling him?
  - If you chose to tell him, what do you think his response would be?
  - If you chose not to tell him, how would you hide it from him?
- [If applicable] If you chose to take it, would you want to tell your casual
  partner(s) you’re taking PrEP?
  - If you chose to tell him, what do you think his response would be?
  - If you chose not to tell him, how would you hide it from him?
- Would you want to tell your friends/family you’re taking PrEP? Tell me more about that.
- Do you think you could remember to take PrEP every day? Tell me more about that.
- People who take PrEP need to go to the doctor every 3 months for a check-up and new prescription. Do you think you could attend all required doctor appointments every 3 months? Tell me more about that.
  - *Transportation*
- Do you think you would remember to refill your prescription every month? What might get in the way of getting refills?
- What are some reasons you might not take PrEP daily?
- What might help you to remember to take PrEP every day?
- As I mentioned, right now a daily pill is the only available medication, however, scientists are conducting studies to find different ways people can take PrEP. They are looking at an injection form, a vaginal ring, or an implant.
  - Would you be interested in PrEP if it were an injection? Tell me more about that.
    - *What would be your concerns about getting an injection?*
    - [If yes] How frequently would you be able to go into a clinic or your doctor’s office to get the injection?
  - A vaginal ring is a flexible ring that you would insert into your vagina for a short period of time. Then you take it out and put in a new one. Would you be interested in PrEP if it were a vaginal ring? Tell me more about that.
    - *What would be your concerns about using the vaginal ring?*
    - [If yes] How frequently would you want to change out the ring?
  - An implant is a small object that would go under your skin that would last for a while. Would you be interested in PrEP if it were an implant? Tell me more about that.
    - *What would be your concerns about getting the implant?*
    - [If yes] How long would you want the implant to last?
- Which of these forms of PrEP, the daily pill, an injection, a vaginal ring, or an implant, would you prefer? Tell me more about that.
- If you took PrEP do you think you would be less likely to use condoms during sex? Tell me more about that.
- If you took PrEP do you think you would have sex with more partners? Tell me more about that.
- If they knew what PrEP was, do you think any of your female friends would take PrEP? Tell me more about that.
- Do you think any of your male friends would take PrEP? Tell me more about that.
- Any last thoughts you want to share with me about PrEP?
- Are you interested in seeing a medical professional to learn more about PrEP?
  - Yes: We are working with CommUnityCare Clinics. [Show them the resource guide with the list of CUC clinics] Here are their clinics, which one is closest to you? Would you like to call them at the end of our interview to set up an appointment with them? I will give you their contact information. I would like to keep in touch to see how your appointment went and to remind you about our next interview in about a month. What is the best way for me to contact you? Text? Phone call? E-mail?
  - No: Tell me more about that. Are there any barriers as to why you do not want to talk to a medical professional about PrEP? Would you consider taking PrEP on your own in the future? I would like to keep in touch and to remind you about our next interview in about a month. What would be the best way for me to contact you? Text? Phone call? E-mail?

Debriefing: Thank you, that is all of the questions I have for you. Do you have any concerns after answering questions? I sincerely appreciate the time you took to answer these questions, and I have $20 for you to thank you for your time and participation [$5 travel and $15 childcare if applicable]. Also, here are some resources for you or someone you know in case you are interested.

**Interview Guide—1-Month**

Questions were similar to baseline interview except asked what has changed from the previous interview.

**Interview Guide—3-Months**

Questions were similar to baseline interview except asked what has changed from the previous interview. We added questions about future PrEP programs.

We are thinking about developing a program to help women take PrEP and we would like to get your thoughts on how we can create a program that would be helpful.

- For a woman who might be at-risk for HIV, what would be some reasons she might want to start taking PrEP?
- Let’s think about [African American/Latina] women in general. What might be some cultural barriers for [African American/Latina] women to taking PrEP? [If race/ethnicity is a sensitive topic, just ask about women of different racial and ethnic backgrounds in general]
- How does stigma play a role in taking PrEP among women?
- What are some things that can help [African American/Latina] women at-risk for getting HIV start PrEP?
  - What are some things that can be done to help them to keep taking PrEP?
- [If they are interested in PrEP] What types of things do you think would make it easier for women such as yourself to take PrEP?
  - What could a program do that could help?
  - How would you design a program to help these women?
- [If they are not interested in PrEP] Although you’re not interested in taking PrEP, for women who might be but don’t know about, what types of things do you think would make it easier to take PrEP?
  - What could a program do that could help?
- What would you want this type of program to look like? (e.g., program structure, length, duration)
- What topics would you include in this type of program?
- Where do you think a program like this should take place?
- Who should be the person to deliver this program [e.g., person from their community, nurse, health educator, etc.?
- What would you name this program?
- Now that our final interview is over, tell me what you thought about participating in this study.
- Is there anything else you’d like to add about PrEP or this study?

Debriefing: Thank you that is all of the questions I have for you. Do you have any concerns after answering questions? I sincerely appreciate the time you took to answer these questions and participating in this study, and I have $35 for you. Also, here are some resources for you or someone you know in case you are interested.

**Check-In Scripts**

**Check-In 1 Text (2 weeks after Interview 1):**

**Participants Interested in PrEP:**

Hi, this is [name] with Layers. I’m just checking in to see how you’re doing. [Wait for response]

Have you had a chance to make an appointment for PrEP yet?

- If yes: When is your appointment?
- If no: What got in the way of you making your appointment?

Your next interview is on [date] at [time]. Does that still work?

- If yes: Great! We’ll see you then!
- If no: Would you like to reschedule now or would you like me to contact you closer to your interview date?
  - Reschedule now: Wonderful, we have these dates and times available [find dates and times that fit with interviewer and participant’s schedules].
  - Reschedule later: I’ll check back in with you in a couple of weeks to see how you’re doing and to reschedule.

If you have any questions before we next chat then let us know!

**Participants Not Interested in PrEP:**

Hi, this is [name] with Layers. I’m just checking in to see how you’re doing. [Wait for response]

Your next interview is on [date] at [time]. Does that still work?

- If yes: Great! We’ll see you then!
- If no: Would you like to reschedule now or would you like me to contact you closer to your interview date?
  - Reschedule now: Wonderful, we have these dates and times available [find dates and times that fit with interviewer and participant’s schedules].
  - Reschedule later: I’ll check back in with you in a couple of weeks to see how you’re doing and to reschedule.

If you have any questions before we next chat then let us know!

**Check-In 2 Text (2 weeks after Interview 2):**

**Participants Interested in PrEP:**

Hi, this is [name] with Layers. I’m just checking in to see how you’re doing. [Wait for response]

Have you had a chance to make an appointment for PrEP yet?

- If yes: When is your appointment?
- If no: What got in the way of you making your appointment?

I’ll check back in with you in a couple weeks to see how you’re doing. If you have any questions before then let us know!

**Participants Taking PrEP:**

Hi, this is [name] with Layers. I’m just checking in to see how you’re doing. [Wait for response]

How’s it going with taking PrEP? [Wait for response]

How many pills did you take over the last 7 days? [Wait for response]

I’ll check back in with you in a couple weeks to see how you’re doing. If you have any questions before then let us know!

**Participants Not Interested in PrEP:**

Hi, this is [name] with Layers. I’m just checking in to see how you’re doing. [Wait for response]

I’ll check back in with you in a couple weeks to see how you’re doing. If you have any questions before then let us know!

**Check-In 3 Text (4 weeks after Interview 2):**

**Participants Interested in PrEP:**

Hi, this is [name] with Layers. I’m just checking in to see how you’re doing. [Wait for response]

Have you had a chance to make an appointment for PrEP yet?

- If yes: When is your appointment?
- If no: What got in the way of you making your appointment?

I’ll check back in with you in a couple weeks to see how you’re doing. If you have any questions before then let us know!

**Participants Taking PrEP:**

Hi, this is [name] with Layers. I’m just checking in to see how you’re doing. [Wait for response]

How’s it going with taking PrEP? [Wait for response]

How many pills did you take over the last 7 days? [Wait for response]

I’ll check back in with you in a couple weeks to see how you’re doing. If you have any questions before then let us know!

**Participants Not Interested in PrEP:**

Hi, this is [name] with Layers. I’m just checking in to see how you’re doing. [Wait for response]

I’ll check back in with you in a couple weeks to see how you’re doing. If you have any questions before then let us know!

**Check-In 4 Text (6 weeks after Interview 2):**

**Participants Interested in PrEP:**

Hi, this is [name] with Layers. I’m just checking in to see how you’re doing. [Wait for response]

Have you had a chance to make an appointment for PrEP yet?

- If yes: When is your appointment?
- If no: What got in the way of you making your appointment?

Your next interview is on [date] at [time]. Does that still work?

- If yes: Great! We’ll see you then!
- If no: Would you like to reschedule now or would you like me to contact you closer to your interview date?
  - Reschedule now: Wonderful, we have these dates and times available [find dates and times that fit with interviewer and participant’s schedules].
  - Reschedule later: I’ll check back in with you in a couple of weeks to see how you’re doing and to reschedule.

If you have any questions before we next chat then let us know!

**Participants Taking PrEP:**

Hi, this is [name] with Layers. I’m just checking in to see how you’re doing. [Wait for response]

How’s it going with taking PrEP? [Wait for response]

How many pills did you take over the last 7 days? [Wait for response]

Your next interview is on [date] at [time]. Does that still work?

- If yes: Great! We’ll see you then!
- If no: Would you like to reschedule now or would you like me to contact you closer to your interview date?
  - Reschedule now: Wonderful, we have these dates and times available [find dates and times that fit with interviewer and participant’s schedules].
  - Reschedule later: I’ll check back in with you in a couple of weeks to see how you’re doing and to reschedule.

If you have any questions before we next chat then let us know!

**Participants Not Interested in PrEP:**

Hi, this is [name] with Layers. I’m just checking in to see how you’re doing. [Wait for response]

Your next interview is on [date] at [time]. Does that still work?

- If yes: Great! We’ll see you then!
- If no: Would you like to reschedule now or would you like me to contact you closer to your interview date?
  - Reschedule now: Wonderful, we have these dates and times available [find dates and times that fit with interviewer and participant’s schedules].
  - Reschedule later: I’ll check back in with you in a couple of weeks to see how you’re doing and to reschedule.

If you have any questions before we next chat then let us know!
