# Supplementary material for: A Qualitative Exploration of PrEP Interests, Barriers, and Interventions Among Black and Latina Cisgender Women in the U.S
Source: Arch Sex Behav. 2023 Oct 5;53(2):771–83. doi: 10.1007/s10508-023-02712-5 (PMC10844362; doi:10.1007/s10508-023-02712-5)
Supplement: Supplementary file 2 — Supplementary file2 (DOCX 26 KB) [file 10508_2023_2712_MOESM2_ESM.docx]

**Supplement B: Codebook**

| **Code** | | | **Term** | **Definition** |
| --- | --- | --- | --- | --- |
| 1 | | | Personal Life, Daily Routine, Living Situation, and Economic Situation |  |
|  | 1.1 | | Daily Life Description | Include anything done on a regular basis (e.g., going to food pantries, attending school) |
|  | 1.2 | | Current Living Situation |  |
|  |  | 1.2.1 | Prior Living Situation | Any living situation, including homeless |
|  | 1.3 | | Income Sources | Including SNAP, TANF |
|  |  | 1.3.1 | Financial difficulty |  |
|  |  | 1.3.2 | Employed |  |
|  | 1.4 | | Supportive Resources |  |
|  |  | 1.4.1 | Emotional Support | Includes family or friends |
|  |  | 1.4.2 | Community Support | Includes community organizations, churches |
|  |  | 1.4.3 | Tangible Support | Food, money, shelter |
|  | 1.5 | | Personal Strengths |  |
|  |  | 1.5.1 | Life Goals |  |
|  | 1.6 | | Ethnic/Racial Culture |  |
|  |  | 1.6.1 | Ethnic/Racial Challenges | Include if they say none |
|  |  | 1.6.2 | Ethnic/Racial Advantages | Include if they say none |
|  | 1.7 | | Legal System | Broad--anything related to the law including CPS, jail or prison, juvenile detention, getting arrested, calling the police on a partner |
|  | 1.8 | | Trauma |  |
|  |  | 1.8.1 | Previous Trauma | Child physical abuse, death of family or friend, assault to family, friend, or participant |
|  |  | 1.8.2 | Childhood Sexual Abuse | Non-consensual sexual contact of any kind when participant is 16 or younger, or consensual sex when participant is 16 or younger and partner is 5+ years older |
|  |  | 1.8.3 | Recent Trauma | Death of family or friend, assault to family, friend, or participant within past 5 years |
|  | 1.9 | | School | Participant or child |
| 2 | | | Relationships |  |
|  | 2.1 | | Steady | Committed relationship such as a husband, fiancé, boyfriend, etc. |
|  | 2.2 | | Casual | Non-committed fling, such as a one-night stand, sex with someone a few times |
|  | 2.3 | | Steady-Casual | Non-committed partner with whom they have sex with regularly over a period of time but there is no relationship |
|  | 2.4 | | Cheating partner | Includes multiple partners |
|  | 2.5 | | Cheating participant | Includes multiple partners |
|  | 2.6 | | Family |  |
|  | 2.7 | | Friends |  |
|  | 2.8 | | Mother |  |
|  | 2.9 | | Father |  |
|  | 2.10 | | Siblings |  |
|  | 2.11 | | In-Laws | Includes boyfriend’s parents |
|  | 2.12 | | Former Partner |  |
| 3 | | | Sexual Experiences |  |
|  | 3.1 | | First Sexual Experience | Non-penetrative |
|  | 3.2 | | First Vaginal Sexual Experience |  |
|  | 3.3 | | Sex & Relationship Dynamics | In general, not necessarily their relationship |
|  |  | 3.3.1 | Initiator of encounter |  |
|  |  | 3.3.2 | Initiator of conversations about safe sex |  |
|  |  | 3.3.3 | Current Sexual & Relationship Dynamics |  |
|  |  | 3.3.4 | Sexual & Relationship Goals |  |
|  | 3.4 | | Transactional sex | Includes survival sex |
| 4 | | | Contraception |  |
|  | 4.1 | | Condom Usage | Includes non-use of condoms |
|  | 4.2 | | Birth Control | Includes getting tubes tied |
| 5 | | | Relationship Challenges |  |
|  | 5.1 | | Verbal/Emotional Abuse | Threats, destroying participants' belongings or inanimate objects, calling participant names or swearing at them |
|  | 5.2 | | Physical Abuse | Pushing, grabbing, choking, kicking, anything that did or could result in an injury |
|  | 5.3 | | Sexual Abuse | Convincing participant to have sex when she says no, sex w/o a condom when she wants to use one, having sex with participant when she's under the influence, having sex with participant when she's sleeping |
|  | 5.4 | | Economic Abuse | Making a participant financially dependent on him, doing anything that forced a participant to lose her job |
|  | 5.5 | | Rape | Forcible sex when participant does not want sex, says no and does not give in to sex; Includes partner or someone else |
|  | 5.6 | | Stalking | Constantly contacting a participant against her will, showing up places unwanted (current or former partner) |
|  | 5.7 | | Social isolation | Keeping a participant from friends or family, disapproving of a participant contacting friends or family |
|  | 5.8 | | Reproductive Abuse | Trying to get a participant pregnant when she does not want to (hiding birth control, poking holes in condom, pretending to put condom on), preventing a participant from getting pregnant when she wants to (can be anyone, not necessary a partner |
|  | 5.9 | | Unhealthy relationship | Interactions between participant and partner that are "red flags" or unhealthy but not to the point of abuse |
|  |  | 5.9.1 | Verbal/Emotional | Conversations that are not verbal/emotional abuse, but partner says something inappropriate that hurts participants' feelings; partner doesn't directly threaten participant, but she is threatened by them |
|  |  | 5.9.2 | Physical | Partner doesn't intentionally hurt participant, but physical interaction (such as play fighting) gets out of hand |
|  |  | 5.9.3 | Sexual | Rough sex that isn't abusive but participant is uncomfortable (she does not tell him), participant asks him to use condom and he gives her a difficult time but eventually he gives in |
| 6 | | | Pregnancy & Children |  |
|  | 6.1 | | Pregnant |  |
|  | 6.2 | | Gave Birth |  |
|  | 6.3 | | Did not give birth |  |
|  | 6.4 | | Children | Separate coding by child |
|  | 6.5 | | Custody | Only children under 18 |
| 7 | | | Substance Use |  |
|  | 7.1 | | Alcohol |  |
|  | 7.2 | | Marijuana |  |
|  | 7.3 | | Other Drugs |  |
|  | 7.4 | | Sex under the Influence |  |
|  | 7.5 | | Dealing | Participant or partner only |
|  | 7.6 | | Problematic substance use | 4+ drinks on 1 occasion (excessive drinking) or drinking daily; marijuana is smoking multiple times a day and/or 14+ days/month. |
| 8 | | | HIV |  |
|  | 8.1 | | Transmission Knowledge |  |
|  | 8.2 | | Prevention Knowledge |  |
|  | 8.3 | | Treatment Knowledge |  |
|  | 8.4 | | Community Perception of HIV |  |
|  | 8.5 | | Felt At-risk for HIV |  |
|  | 8.6 | | HIV Testing |  |
| 9 | | | HealthCare |  |
|  | 9.1 | | Insurance |  |
|  | 9.2 | | Service Provider | Where the participant goes if sick or injured or any time, they see a medical provider |
|  | 9.3 | | Treatment Adherence |  |
|  | 9.4 | | Mental Health | Include substance use (treatment) |
|  | 9.5 | | Access to services | (Lack of access, difficulty navigating, lack of availability) |
|  |  | 9.5.1 | Access to transitional housing/shelters |  |
|  |  | 9.5.2 | Access to social services | Other than housing |
|  |  | 9.5.3 | Access to mental health services |  |
|  |  | 9.5.4 | Access to healthcare |  |
| 10 | | | PrEP |  |
|  | 10.1 | | PrEP awareness |  |
|  | 10.2 | | Community PrEP Knowledge |  |
|  | 10.3 | | PrEP Perception |  |
|  |  | 10.3.1 | PrEP Concerns |  |
|  |  | 10.3.2 | Perceived PrEP Advantages |  |
|  | 10.4 | | PrEP Disclosure |  |
|  |  | 10.4.1 | Steady Partner | Includes steady-casual partners |
|  |  | 10.4.2 | Casual Partner |  |
|  |  | 10.4.3 | Friends/Family |  |
|  | 10.5 | | PrEP Adherence |  |
|  | 10.6 | | PrEP Appointments |  |
|  | 10.7 | | Interest Level |  |
|  |  | 10.7.1 | High |  |
|  |  | 10.7.2 | Medium |  |
|  |  | 10.7.3 | Low |  |
|  |  | 10.7.4 | None |  |
|  | 10.8 | | PrEP Type |  |
|  |  | 10.8.1 | Injectable |  |
|  |  | 10.8.2 | Vaginal Ring |  |
|  |  | 10.8.3 | Implant |  |
|  |  | 10.8.4 | Pill |  |
|  |  | 10.8.5 | PrEP Preference | Preferred method of taking PrEP |
|  | 10.9 | | Behavior Change on PrEP |  |
|  |  | 10.9.1 | Condom Usage | Use condoms less |
|  |  | 10.9.2 | Sex Partners | Have more partners |
|  | 10.10 | | Friends take PrEP |  |
|  | 10.11 | | Conspiracy Theory/Medical Mistrust |  |
| 11 | | | Intervention |  |
|  | 11.1 | | Content | Content that should be included in the intervention |
|  | 11.2 | | Dose | How many session |
|  | 11.3 | | Length | Duration of intervention |
|  | 11.4 | | Type |  |
|  |  | 11.4.1 | Structural | Anything not at the individual or one-on-one level |
|  |  | 11.4.2 | Social |  |
|  |  | 11.4.3 | Individual |  |
|  | 11.5 | | Location |  |
|  | 11.6 | | Name |  |
|  | 11.7 | | Presenter |  |
|  | 11.8 | | Perception of Current Study |  |
